# A Patient-Centered Forensic Nursing Model of Care for Victims of Law Enforcement Violence

**DOI:** 10.1089/heq.2023.0270

**Published:** 2024-09-17

**Authors:** Maija Anderson, Jacqueline Callari-Robinson, Margaret Glembocki, Elizabeth Louden

**Affiliations:** ^1^ Nursing Department, in the School of Community Health and Policy, Morgan State University, Baltimore, Maryland, USA.; ^2^ Forensic Nurse for University of Maryland Baltimore Washington Medical Center, Glen Burnie, Maryland, USA.; ^3^ College of Nursing at University of Wisconsin-Milwaukee, Milwaukee, Wisconsin, USA.; ^4^ Department of Natural Sciences, College of Arts and Sciences at Lawrence Technological University, Southfield, Michigan, USA.; ^5^ Department of Nursing at Fitchburg State University, School of Health and Natural Sciences, Fitchburg, Massachusetts, USA.; ^6^ School of Nursing at Nipissing University, North Bay, Ontario, Canada.

**Keywords:** law enforcement violence, police brutality, victims, patient-centered model of care, trauma-informed care, intersectionality, levels of racism, social determinants of health, emancipatory nursing praxis, forensic nursing, nursing, forensic nursing model of care

## Abstract

**Background:**

The manuscript examines the nature, manifestations, and potential causes of law enforcement violence as well the need for a model of care for victims. Specifically, it explores development of a preliminary forensic nursing model of care. The questions posed over the course of development of the model follow (1) What are the challenges to developing a rudimentary forensic nursing model of care for victims of law enforcement violence? (2) What are the tenets to be utilized in developing the model? (3) What additional recommendations are to be considered in refining and expanding the model?

**Key Concept:**

A review of the literature in forensic nursing found a gap in care for victims of law enforcement violence. To address the gap given the lack of research, a preliminary model of care was developed based on key constructs from the following established models: (1) Theory of Abolition, (2) Critical Race Theory, (3) Levels of Racism, (4) Intersectionality, (5) Social Determinants of Health, (6) Emancipatory Praxis – Theory of Forensic Nursing, (7) Trauma-Informed Model of Care, and (8) Patient-Centered Model of Care.

**Implications for practice:**

The preliminary model developed adheres to the International Council of Nurses guidelines, which emphasize the nurse’s duty to care without judgment or bias. Protocols established must be followed precisely to mitigate potential conflicts of interest in care of the victim. A practical application algorithm was developed based on care provided to other victims of violence.

**Conclusion:**

The model developed was focused on forensic nursing care. There is a need for further refinement involving an interdisciplinary approach. There is also a need for additional research as it relates to forensic nursing’s role in caring for victims of law enforcement violence.

## Reflections from the Authors

As a woman and nurse of color, having the opportunity to care for victims of law enforcement violence (LEV) was an enlightening experience. Prior to my own interaction with law enforcement, where excessive force was utilized, I was disengaged from the patients to some extent. I was not yet ready to advocate for them; I wanted to but did not know how. What I have learned along the way is that there is a need for a model of care to serve as a guideline for establishing consistency in care areas where LEV victims may present. As I have evolved in my career, I am more aware of the type of advocacy that I can provide, both at the micro and macro levels, in support of different outcomes for victims of LEV. The micro level involves serving as their advocate in the acute phase, establishing a rapport with the patient, removing any bias from the encounter, collecting evidence the way that I have been trained to do for other victims of violence, and ensuring their safety during the process. The macro level involves advocating for victims of LEV within the discipline of forensic nursing, with other professionals who care for or engage victims of LEV, developing a rudimentary forensic nursing model of care, and legislation supporting the care of victims of LEV.

As a White nurse and woman caring for victims and patients, I had the privilege and was uniquely positioned to care for victims and patients who had experienced violence over the past 30 years. I, therefore, again, through my privilege as a White nurse, had opportunities available to me to create medical forensic programs in Wisconsin and throughout the United States. Our care was victim centered, although when we cared for patients who were identified as suspects by law enforcement, we did not offer the same comprehensive and compassionate care we provided to victims and patients per protocol. I became complicit in the ideology of forensic nursing, the binary belief of victim-perpetrators, and not recognizing the complexity of life in which victims can be perpetrators and perpetrators can be victims of crime. Through my practice and service on a forensic nursing social justice committee, I observed how structural racism resulted in the dehumanization of patients regardless of whether they were identified as victims or suspects by the health care and criminal justice systems in their response to patients and victims. The Social Justice Committee’s leadership inspired profound discomfort, leading to deep reflection and awakening. I began my antiracist journey, for instance, attending sessions on reckoning racism in nursing, attending conferences, listening, journaling, being conscious of my privilege, and not taking up too much space, making space for nurses of color. I believe that patients suffering from LEV need forensic nursing care, and I want to provide patients suffering from LEV with empathetic, holistic, and compassionate patient-centered care per protocol.

As a Canadian, White woman, and nurse with many years of experience at the bedside, I was fortunate to have an opportunity to visualize what collaboration and education brought to interprofessionalism—in this case, law enforcement and nursing. On exiting a long-term role that provided examples of how collegiality could be successful, I discovered that it was not the case elsewhere and was disheartened to realize the yawning gap that existed quite profoundly and very needlessly. It is my privilege to join my colleagues in advancing societal reform to ensure appropriate care, a basic human right, for victims of violence.

Many of us, members of the Social Justice Collaborative, were originally forensic nurses working in emergency departments in the late 1980s and the 1990s. Through the narrow and disconnected lens of providing compassionate and patient-centered care, we believed at the time, although altruistically and idealistically, that we were answering the call of suffering to diverse victims and patients. Patients and victims who were experiencing violence experienced pain, injury, fear of infections, and poor outcomes at the hands of violence. We also observed secondary revictimization, narrow focus, and disjointed health care and criminal justice responses to patients and victims and believed that we could provide a more empathetic and compassionate response to victims and patients.

To resolve this issue, we started proliferating programs designed to provide round-the-clock on-call care in urban and rural communities by partnering with advocates and law enforcement, without the benefit of scholarly support within nursing. Consequently, we were disconnected from the realities hidden within our close alignment and relationship with the ideology of the criminal justice system and the impact of forensic nursing care on our patients, relatives, communities, and society at large. Forensic nursing education needs to be grounded in research that acknowledges that life is complicated and that we serve victims and perpetrators who are impacted by structural racism and the impact of violence. We must advance holistic quality and equity of care to focus on the health care needs of victims and patients instead of operating and existing to improve the quality and legal admissibility of evidence.

## Introduction

Law enforcement violence (LEV) is not a new phenomenon; rather, it is increasingly being brought to public attention as a result of advances in technology that allow for the recording and broadcasting of violent incidents. For instance, George Floyd’s death due to LEV inflicted by Minneapolis police officers caused worldwide outrage, precipitating protests that demanded police reform and increased accountability. The George Floyd video and its aftermath seem to be the catalyst for moving forward with much-needed reforms within LEV. Professional health care associations have contributed to the conversation by publishing statements that denounce racism and violence and are in support of reducing the prevalence of LEV through advocacy, education, legislation, and research.^1–6^

Mitigating adverse health outcomes related to LEV at the community level requires additional action from the health care community, as there is an understanding that LEV is a public health issue that results in death, injury, trauma, and stressors that disproportionately impact communities of color. While the true impact of such acts is difficult to quantify, it is understood that the available data establish a correlation between LEV and adverse health outcomes.^4^

According to research, minorities are subject to negative stereotypes and experience prejudice by law enforcement officials and health care providers, resulting in mass numbers of lives lost to LEV.^7^ The data that we do have available to us support the crucial need for a model of care specifically designed to address the needs of LEV victims.

As nurses, the charge or call to action relative to this issue is embedded within our code (s) of ethics. Components of the International Code of Ethics for Nurses, American Nurses Association, and the International Association of Forensic Nursing germane to this construct are shown (verbatim) in Table 1.^8–11^

**Table 1.** Call to Action

International Code Of Ethics for Nurses^8^	*Element 1.* Nurses and people—the nurse’s primary professional responsibility is to people requiring nursing care. The nurse shares with society the responsibility for initiating and supporting action to meet the health and social needs of the public, in particular, those of vulnerable populations. The nurse advocates for equity and social justice in resource allocation, access to health care, and other social and economic services.
American Nurses Association (ANA) Code of Ethics^9^	*Provision 1*—The nurse practices with compassion and respect for the inherent dignity, worth, and unique attributes of every person. *Provision 2*—The nurse’s primary commitment is to the patient, whether an individual, family, group, community, or population. *Provision 3*—The nurse promotes, advocates for, and protects the rights, health, and safety of the patient. *Provision 8*—The nurse collaborates with other health professionals and the public to protect human rights, promote health diplomacy, and reduce health disparities. *Provision 9*—The profession of nursing, collectively through its professional organizations, must articulate nursing values, maintain the integrity of the profession, and integrate principles of social justice into nursing and health policy.
International Association of Forensic Nurses (IAFN) Vision of Ethical Practice^10^	*Responsibility to the Public*—Forensic nurses have a professional responsibility to serve the public welfare. Forensic nurses should be actively concerned with the health and welfare of the global community. Forensic nurses should recognize their role in preventing violence, which includes understanding the societal factors, such as oppression that promotes violence. Forensic nurses acknowledge the value and dignity of all human beings and strive to create a world where violence is not accepted.
International Association of Forensic Nurses (IAFN) Position Statement: Violence is a Health Issue^11^ *Position Paper:* *Violence Is a Public Health and Health care Issue (2022*)^a^,^10^	*Association Position 1*—Forensic nurses have a professional and ethical responsibility to serve, advocate for, and empower patients, families, and their communities. *Association Position 2*—Forensic nurses organize and participate in facilitating the development of policies and procedures that foster the implementation of prevention and intervention programs in response to violence. *Association Position 4*—Forensic nurses have the opportunity to improve the ultimate health outcomes that result from violence as forensic nurses are uniquely positioned in intersecting systems such as health care, community, and legal environments for early identification of patients at risk of victimization or perpetration of violence. *Association Position 6*—Forensic nurses are able to establish and promote identification, intervention, and prevention programs, with the recognition that sustained societal change requires action that includes support of research, development of public policy, and passage of legislation to effectively reduce and eliminate the causes, consequences, and costs of violence.

^a^ IAFN Position Paper: Violence Is a Public Health and Health care Issue is also listed in the table as the concepts are similar to what was articulated in the 2009 position statement.

Acronyms and terms utilized in the article along with definitions where necessary are outlined (verbatim) from their resources in Table 2.^12–24^

**Table 2.** Terms, Acronyms, and Definitions

Term/acronym	Definition
ACEP	American College of Emergency Physicians
AMA	American Medical Association
American Nurses Association Code of Ethics for Nurses	“A guide for carrying out nursing responsibilities in a manner consistent with quality care and the ethical obligations of the nursing profession.”
ANA	American Nurses Association
APHA	American Public Health Association
Chain of Custody	The U.S. Department of Justice defines chain of custody as “documentation that establishes a record of control, transfer and final disposition of evidence in a case.”^12^
Critical Race Theory	“The practice of interrogating the role of race and racism in society that emerged in the legal academy and spread to other fields of scholarship. It critiques how the social construction of race and institutionalized racism perpetrates a racial caste system that regulates people of color to the bottom tiers. It also recognizes that race intersects with other identities including sexuality, gender identity, and others. It recognizes that racism is not a bygone relic of the past, but instead acknowledges that the legacy of slavery, segregation, and the imposition of second-class citizenship on Black Americans and other people of color continues to permeate the social fabric of this nation.”^13^
Constructed Theory of Forensic Nursing Care	“This theory affirms the focus of forensic nursing is on the nurse-patient relationship and improved health outcomes. Additional results of forensic nursing care are improved forensic science and criminal justice system outcomes.”^14^
Feminism	“Belief in and advocacy of the political, economic, and social equality of the sexes expressed especially through organized activity on behalf of women’s rights and interests.”^15^
Feminist Theory	This theory views the social world in a way “that illuminates the forces that create and support inequity, oppression, and injustice and in doing so promotes the pursuit of equality and justice.”^16^
Forensic Nursing	“The practice of nursing globally when health and legal systems intersect.”
Hegemony	“The dominance of certain ideologies, beliefs, values, or views of the world over other possible viewpoints.”^17^
IAFN	International Association of Forensic Nurses
ICN	International Council of Nurses
Internalized Racism	“Acceptance by members of the stigmatized races of negative messages about their own abilities and intrinsic worth.”^18^
Institutionalized Racism	“Polices, rules, practices, etc. that have become a usual part of the way an organization or society works, and that result in and support a continued unfair advantage to some people and unfair or harmful treatment of others based on race.”^18^
Intersectionality	“The complex, cumulative way in which the effects of multiple forms of discrimination (such as racism, sexism, and classism) combine, overlap, or intersect especially in the experiences of marginalized individuals or groups.”^19^
Law Enforcement Violence (LEV)	Refers to “acts of violence incited on individuals of law enforcement at any level or law enforcement agency type, while in their respected role of duty. These acts of violence include physical, psychological, sexual, and the act of neglect. This term is being utilized instead of police brutality because it encompasses all branches of law enforcement versus one.”^4^
Levels of Racism—A Gardener’s Tale	“A framework for understanding racism on the levels of institutionalization, personally-mediated and internalized which is useful for raising hypothesis about the basis of race associated differences in health outcomes as well as effective interventions to eliminate the differences.”^18^
NLN	National League for Nurses
Patient-Centered Model of Care	“A care model that is respectful of and responsive to individual’s patient preferences, needs and values, and ensures that patient values guide all clinical decisions.”^20^
Personally Mediated Racism	“Prejudice and discrimination, where prejudice means differential assumptions about the ability, motives, and intentions of others according to their race.”^18^
Social Determinants of Health	“The conditions in the environment where people are born, live, learn, work, play, worship and age that affect a wide range of health, functioning, and quality of life outcomes and risks.”^21^
Theory of Emancipatory Nursing Praxis	“A middle range theory describing the transformative learning process nurses experience as they come to know and engage in social justice as allies.”^22^
Trauma Informed Care	“Care that acknowledges the need to understand a patient’s life experiences in order to deliver effective care and has the potential to improve patient engagement, treatment adherences, health outcomes, and provider and staff wellness that avoids re-traumatization of the patient.”^23^

## Review of Literature

Recent literature demonstrates that social trust is directly connected with confidence in law enforcement and that trust is influenced by persistent socioeconomic indicators. It is estimated that ∼1,000 civilian lives are taken each year by law enforcement officers in the United States, and Black men are 2.5 times more likely than White men to be killed by law enforcement during their lifetime. Police violence against civilians is a leading cause of death in the U.S. for all young men, but specifically young Black men.^25^ Actual prevalence is not known due to a lack of official data; however, we do know that there is a persistent and disproportionate trend of fatal police shootings among Black, Hispanic, and American Indian (AI) populations. These violent encounters have a profound impact on health, the community, and individual opportunities. Edwards et al. observed that structural inequalities between White people and people of color are seen in the policing system; more people are killed in the United States by police than in any other advanced democracy. Moreover, people of color face a higher likelihood of being victims of police violence than White men do, leading to early mortality. This risk peaks in young adulthood (age 20–35), yet men of color face a lifetime risk of being killed by the police. Black men and women, American Indian, and Alaska Natives are significantly more likely to die from excessive police force than White men and women.^25^

Black communities in urban metropolitan areas are disproportionately affected by violence when experiencing tensions with law enforcement. Recent acts of police brutality captured on videos and circulated widely on social media have significant implications for community relations with law enforcement. The inherent fear that members of the Black community embody about police officers potentially using excessive and unjustified force in policing can deter them from seeking law enforcement assistance.^26^ Accordingly, there is a level of powerlessness experienced by people of color when interacting with law enforcement that stems from a history of police violence and is further exacerbated by recent high-profile acts of violence by law enforcement caught on video. This powerlessness is compounded by the current and historical trauma experienced by people of color for generations, which often embeds a lack of trust in law enforcement within these communities.^27^

While a large amount of research concerning LEV focuses on the male experience, it is important to recognize that since 1970, the rate of women incarcerated in the United States has increased by a factor of 14. Crenshaw reported the highest growth rate of any incarcerated population.^28^ A breakdown of the data indicates that the majority of women criminalized and incarcerated were Black, Indigenous, and Latinx. Between 1986 and 1991, the number of Black women imprisoned for drug offenses increased by more than 800%, nearly thrice that of White women. By 2003, Indigenous women were reportedly incarcerated at a rate of 6.7 times that of White women.^29^ A report released by the Center for Intersectionality and Social Policy Studies of “routine” traffic stops in New York City in 2013 found that the rate of racial disparities in stops and arrests is identical among men and women. Rates for women stopped based on race as follows: 13.4% White, 53.4% Black, and 27.5% Latinx. To provide an additional context, U.S. Census Data at the time indicate that 27% of New York City residents were Black yet comprised 53% of stops.^29^

It is also important to recognize the issue of sexual assault (SA) and the harassment of women in the hands of law enforcement. Although there are few official data outlining the extent of sexual harassment and assault by members of law enforcement, news reports and existing data indicate that these behaviors occur frequently.^30–32^ A letter to the President’s Task Force on 21st-Century Policing details the findings of a review of police misconduct.^28^ The letter highlighted findings from The Cato Institute’s National Police Misconduct Statistics and Report Project, which found that officer-involved sexual misconduct was the second most common form of misconduct reported.^33^ Women, particularly those who are Black, Indigenous, women of color, minors, immigrants, lesbian, transgender, homeless, and low income, are most vulnerable to sexual misconduct perpetrated by law enforcement. These actions contribute to the reluctance to seek medical care. Consequently, for Black and AI women who are survivors of SA, it is reported that they are less likely to have their injuries treated, receive follow-up care, be provided information on HIV, and/or have contact with SA treatment centers compared with White women.^34,35^

Moreover, a comparison of global data articulates that the United States has, in comparison to other nations, an inflated presence of law enforcement.^36^ Being roughly the same area size but with a population that is nine times greater than that of Canada, the United States hosts 18,000 law enforcement agencies, including local, state, and federal police forces. Canada, which administers police at the municipal, provincial, and federal levels, has fewer than 200 police services; as of May 15, 2019, 4% of police officers and 3% of recruits self-identified as Indigenous.^37^ In the 2016 Canadian census, Indigenous people represented 5% of the total Canadian population. However, the census also showed that 8% of police officers and 11% of recruits in Canada were visible minorities. Individuals designated as visible minority populations represented 22% of Canada’s population in the 2016 census.^38^

In attempting to determine why the United States leads world democracies in police killings, researchers touched on funding, training, arming, and disciplining. Moreover, while most advanced democracies approach their law enforcement system differently than the United States, many also struggle with police brutality and tense relations between law enforcement and minority communities.^36^ From a North American perspective, law enforcement has a superior advantage as they carry firearms, while police officers in the United Kingdom (with the exception of Northern Ireland) are not armed as they police by consent, meaning they rely on the public to respect their authority.

While there remains a paucity of reliable data on the extent of the impact of law enforcement on health outcomes, a synopsis of the available data indicates that LEV and misconduct jeopardize the health and safety of individuals, families, and communities, disproportionately impacting people of color, immigrants, gay, lesbian, and transgender people, as well as those of lower socioeconomic status.

## Restorative Justice Undergirds Principles Germane to Health Care and Nursing

“Restorative justice represents a complete paradigm shift from viewing harm as a violation of the law to understanding it as a violation of people and relationships that requires accountability and healing,” says Ashlee George, associate director of Impact Justice’s Restorative Justice Project. She goes on to explain: “This approach gives all communities, especially marginalized ones, a powerful tool to replace the criminalization of youth of color.” Restorative justice in health care represents a powerful commitment to healing and humanism, both of which lie at the heart of nursing practice.

Specifically, understanding restorative justice as it applies to the role of forensic nurses in caring for victims of violence is crucial in caring for victims of LEV during times of crises. Forensic nurses provide direct patient care to individuals, families, communities, and systems that have experienced violence or trauma.^39^ In the review, “An Integrative Review of Nurse Authored Research to Improve Health Equity and Human Rights for Criminal Justice Involved People,” Goshin and colleagues sound a call to action to forensic nurses where restorative justice principles are the underpinnings of this care.^40^ Forensic nurses’ attitudes, beliefs, and knowledge about police violence toward civilians and acceptance of this form of violence can shape public response, consequently having a negative influence on victims physical, emotional, and psychological health. Forensic nurses advocate for health care justice by providing leadership and support in changing attitudes, policies, and health practices to promote the healing of victims of violence. For survivors of violence, positive intervention with police and health care, including forensic nurses, could make a difference between life and death.^41^

We argue that forensic nursing care for victims of police violence should be addressed at local, state, and federal levels. Systematic changes are needed regarding access to forensic nursing care for victims of this type of violence. Ultimately, forensic nurses must advocate legislation that supports the nursing care of individuals victimized by LEV.

Exploration of how restorative justice can be partnered with the criminal justice system in the provision of a patient-centered forensic nursing care model for the health care response will support equity in health care, and its impact on outcomes for victims of LEV should be explored. We argue that such a partnership would be a primary preventive strategy in which forensic nurses are involved in the education and training of law enforcement officers in areas related to poor health outcomes related to LEV (e.g., use of choke holds, tasing, and use of body weight).

## Recommendations

Understanding that LEV against citizens is a global public health concern that must be addressed, we encourage a patient-centered model of care. We argue that forensic nurses are positioned, through knowledge and commitment, to offer leadership in creating safer social and health systems for all individuals by working within interprofessional systems to develop a way of building trust between vulnerable populations, law enforcement, and the health care system.

As a profession, forensic nursing must commit to the development and implementation of evidence-based guidelines for the care of patients affected by LEV. Forensic nurses are obligated to ensure access to competent, quality-based care within the criminal justice system as health care partners providing for the human rights and health care of those in community-based custody in the following ways:

- Examine and identify gaps in both understanding and responding to race-related and other forms of violence toward and among our patients, within the profession, and with community partners.
- Strategies to increase the diversity of forensic nursing and leadership in forensic nursing organizations to better represent the populations served.
- Educate fellow forensic nurses about the principles of restorative justice by actively seeking opportunities to listen, learn, and collaborate with diverse populations and organizations.
- Advocate a forensic nursing response that ensures the safety of victims seeking care for race-related and other forms of violence.

Because forensic nursing is patient-centered care provided to all populations affected by violence, forensic nurses practice impartiality and neutrality.^8^ This framework positions forensic nurses to recognize the value of civic responsibility and accountability toward individuals of diverse communities who are disproportionately targeted by law enforcement. Moreover, a broader understanding of racial and ethnic divisions that reinforce negative stereotypes can help elucidate the societal response to influence structural positivity. This leads to an examination of the tenets required to develop an effective model of care in support of victims of LEV.

## Developing Tenets of the Model of Care

Several models were reviewed to determine which constructs are best suited to form the tenets for the *Patient-Centered Forensic Nursing Model of Care for Victims of Law Enforcement Violence.* These constructs were extracted from the following eight established models: (1) Theory of Abolition, (2) Critical Race Theory (CRT), (3) Levels of Racism—a Theoretical Framework and a Gardener’s Tale, (4) Intersectionality, (5) Social Determinants of Health, (6) Emancipatory Praxis—Theory of Forensic Nursing, (7) Trauma-Informed Model of Care, and (8) Patient-Centered Model of Care. A synopsis of the models and germane constructs for the proposed model follows.

### The Theory of Abolition

The theory of abolition at its core involves constructing a society without imprisonment and policing. The vision of this new society requires dismantling oppressive institutions or systems, building new ones, and embracing transformation. Davis describes disconnects in our society, wherein common-sense assumptions define a prison system as “imprisonment as a fate reserved for others, a fate reserved for the evildoers.”^42^ Davis describes the binary idea of victims and evildoers, and this may be the ideology of forensic nursing. In this model, forensic nursing education and care do not center on the truth that life is complicated, and people are both victims and perpetrators.

It is of utmost importance that forensic nursing education addresses the profound impacts of poverty and racism and the intricate nature of the lived experiences of individuals, families, and communities affected by these issues. In her book *Torn Apart*, Dorothy Roberts critically analyzes poverty criminalization and how colonial norms produce racial and class disparities and inequity.^43^

The model of care for LEV victims is a vision created by the Nursing Social Justice Collaborative and an invitation to all practicing forensic nurses to consider how the history and theory of abolition inspired nursing toward our goal of forensic care equity. Abolition, specifically for this article, refers to the works of Frederick Douglass, Angela Davis, and Dorothy Roberts, inviting us to reflect on the abolition of slavery, the prison system, and the child welfare system that perpetuates the continuation of institutional racism, and how it impacts our patient population. This discussion can identify the required research, build collaboration, and articulate a vision for forensic nursing and all victims and patients who experience LEV.

### Critical Race Theory

CRT was developed in the 1970s and the early 1980s based on the philosophical writings of Derrick Bell, Alan Freeman, and Richard Delgado. The theory was developed as a result of various legal scholars, lawyers, and activists noting that many of the advances in the civil rights era were being stifled and, in some cases, even reversed. CRT also draws from the disciplines of philosophy, history, psychology, social science, and law, and its theoretical underpinnings are drawn from legal studies, critical theory, feminist theory, and postmodernism. It also traces the concept of racism in the United States by considering the nation’s legacy of slavery, the Civil Rights Movement, and more recent events.^44^ The writings that inform CRT include those of Sojourner Truth, Frederick Douglass, W.E.B. DuBois, and Martin Luther King Jr.

Delgado and colleagues describe CRT as the notion that racism is a social construct that is not just a byproduct of individual bias or prejudice but is also systemically embedded in legal systems and policies. CRT acknowledges that victims of systemic racism are affected by cultural perceptions of race, and the theory provides them with a way of representing themselves that counters prejudice. CRT has expanded beyond its foundations in legal studies in the fields of education, political science, American studies, ethnic studies, and health care. New studies that have drawn from CRT include Latino critical race, Asian American critical race, American Indian critical race, and critical queer studies.

### Levels of Racism—a Theoretical Framework and a Gardener’s Tale

Jones describes the variable of race as a “rough proxy” for the variable’s socioeconomic status, cultures, and genes that provides an accurate social classification of a people in a race-conscious society.^18^ The author explains race as “not a biological construct that reflects innate differences, but as a social construct that precisely captures the impacts of racism.”^45–47^ The framework describes racism on three levels as follows: institutionalized (also referred to as systemic), personally mediated, and internalized, and how it impacts health outcomes.

Institutionalized racism is described as normative, that is, derived from a standard or norm, particularly that of a behavior. Jones went on to describe it as being structural, as it had been codified in institutions by way of custom, practice, and law for so long that there was no need for an identifiable perpetrator. In situations where LEV is involved, it manifests as a lack of access to power for victims and their loved ones. Examples include differential access to information (e.g., rights as a victim of LEV), resources (e.g., services available to victims of other types of violence), and voice (e.g., representation in government, police oversight, and representation in the media).^18^

Personally mediated racism is described as “prejudice and discrimination, where prejudice refers to differential assumptions about the abilities, motives, and intentions of others according to their race, and discrimination means differential actions toward others according to their race.”^12^ This type of racism can be intentional or unintentional (implicit bias), and may also include acts of commission and/or omission. Personally mediated racism manifests as a lack of respect (e.g., no model of care that addresses victims of LEV and lack of communication with regard to treatment options), suspicion (e.g., considering patients presenting for care in custody of law enforcement as criminals versus patients), devaluation (e.g., surprise that victims of LEV may be something other than criminals), scapegoating (i.e., victims of LEV deserved whatever they got because they did not comply), and dehumanization (e.g., considering LEV toward patients as acceptable behavior).^18^

Internalized racism is described as a form of acceptance by members of stigmatized races of negative messages about their abilities and intrinsic worth. It may manifest as marginalized victims of violence who do not believe in themselves or others who look like them. Black, Indigenous, and people of color may also learn to “accept limitations to one’s own full humanity” to include “diminishing dreams of achievement, right to self-determination, and one’s range of allowable self-expression.”^18^ An example of this is when Black parents feel the need to teach their children about the extra steps to take when engaging with law enforcement to stay alive.

### Intersectionality

Intersectionality describes and speaks to the root causes of power dynamics and analyzes the intersectional nature of oppression and how it impacts the socioeconomic realities of people of color, and, ultimately, how these intersections inform the social determinants of health. Intersectionality theory allows for the analysis of how bylaws, rules, and practices are reproduced in government and become economically, culturally, and societally normative.^48^ Intersectionality also allows for an analysis of the complex nature of these norms by allowing for a reflection on the overlapping forms of discrimination and racialized and gendered violence at the hands of police.^49^

### Social Determinants of Health

**FIG. 1.**
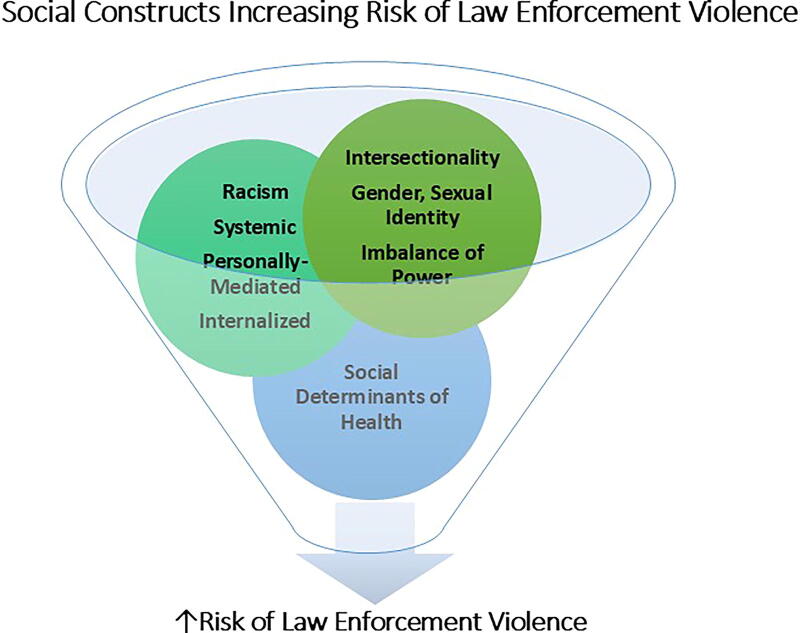
Social constructs increasing risk of law enforcement violence.

Social determinants of health, well-being, and safety include the neighborhoods in which people live, access to healthy food or lack thereof, housing stability, the quality of education, and the ability to access transportation. Scientific evidence proves that disparities in health and health care are based on socioeconomic factors and impact the quality of an individual’s life.^50^ Structural racism, including residential segregation, limits communities’ access to safe neighborhoods, quality education, employment, transportation, and quality of food. According to Bailey et al., structural racism is “not simply the result of private prejudices held by individuals, but is also produced and reproduced by laws, rules, and practices, sanctioned and even implemented by various levels of government, and embedded in the economic system as well as through cultural and societal norms.”^51,52^ It manifests in segregated communities’ fear of law enforcement and is a predictor of the people of color/White disparity in fatal police shootings.^53^ Confronting racism therefore requires not only changing individual attitudes but also transforming and dismantling the policies and institutions that undergird the U.S. racial hierarchy.^52^ Figure 1 illustrates how the confluence of the identified constructs increases the risk of LEV.

### Theory of Emancipatory Nursing Praxis and Forensic Nursing

**FIG. 2.**
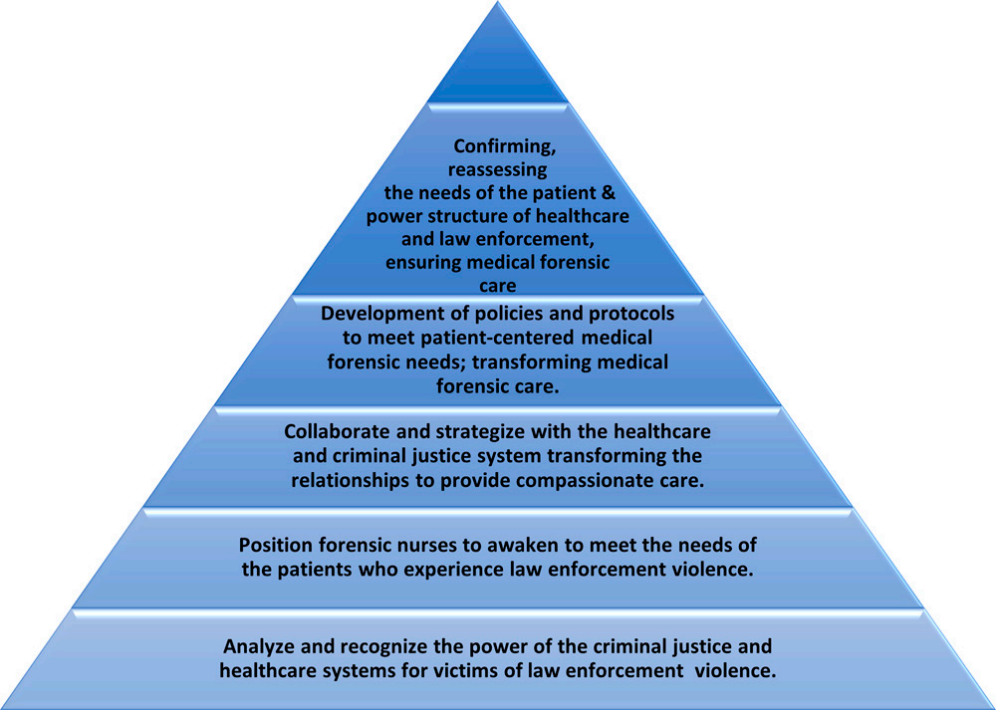
Emancipatory nursing praxis/forensic nursing pyramid (developed with permission of Dr. Roin Walter).

Two middle-range nursing theories center on forensic nurses’ professional roles in patient-centered care and address social justice inequities. Historically, forensic nursing, the integrated practice model for forensic nursing science, has guided forensic nursing’s roles, responsibilities, education, and practice. As forensic nursing evolved into a more emancipatory and comprehensive caring approach, middle-range nursing theory focused on the inclusion of goals concentrated on “ensuring the inherent rights of all people.”^14^ Forensic nursing middle-range theory guides forensic nurses in the care of victims of LEV, enacting their professional responsibility to serve the individual, family, community, and society at large. The emancipatory nursing praxis grounded theory provides forensic nurses with an understanding and action-based process (becoming, awakening, engaging, and transforming) and two conditional contexts (relational and reflexive) to guide forensic nurses’ practice. Figure 2 outlines the practical application of emancipatory nursing praxis to forensic nursing practice.

### Trauma-Informed Model of Care

Trauma is broadly defined as experiences that produce long-lasting emotional pain and distress and contribute to negative health outcomes.^54^ The Substance Abuse and Mental Health Services Administration describes trauma as a widespread public health problem that is harmful and costly. There are several contributing factors, including violence, abuse, neglect, loss, disaster, and other emotionally damaging experiences. Trauma is an “equal opportunity offender” and impacts everyone without regard to age, gender, socioeconomic status, race, ethnicity, geography, or sexual orientation.^23^

Trauma-informed care (TIC) is based on an understanding of the ubiquitous nature of trauma that allows for the development of environments conducive to healing and recovery rather than practices or services that may unintentionally retraumatize victims.^55^ The approach taken is a holistic approach that is cognizant of all components of the patient in need of services. TIC is grounded in an approach that assumes that more often than not, an individual is likely to have a history of trauma and calls for a change in organizational culture, where “emphasis is placed on understanding, respecting, and appropriately responding to the effects of trauma at all levels.”^55^

The TIC recognizes the presence of trauma symptoms and acknowledges the role that traumatic events play in the lives of its victims. Crucial to TIC is a systemic paradigm shift from thinking or inquiring: “What is wrong with this person, or what did they do wrong?” to “What has happened to this person?”^55^ Thus, the intent of TIC is not to treat symptoms or issues related to trauma but to provide adequate and accessible health services to the patient without retraumatizing them. There are five guiding principles of TIC that are constructs for engaging with trauma patients—victims of LEV—to mitigate the risk of retraumatization, including safety, choice, collaboration, trustworthiness, and empowerment.

The Institute of Trauma and Trauma-Informed Care identifies key elements of TIC that are germane to the care of victims of LEV by constructing (or creating) an environment that affords physical and emotional safety, establishing trust and boundaries, supporting patient autonomy and choice, and creating collaborative relationships and opportunities to participate in care.

### Patient-Centered Model of Care

The Institute of Medicine describes a patient-centered model of care as one that is “respectful of and responsive to individual patient preferences, needs, and values and ensures patient values guide all clinical decisions.”^20^ The four C’s of patient-centered care are as follows: Culture, Care, Communication, and Collaboration. The constructs derived from those tenets are germane to the proposed model of care for victims of LEV and are as follows:

- Health care is respectful of all patients’ values, preferences, and needs. Patients should feel comfortable expressing their views, be involved in decision making according to their preferences, and receive respectful care.
- Health care that is coordinated and integrated involves the timely transfer of up-to-date patient information to other health care professional team members and efficient transition of patients between health care settings.
- Patient information is clear, accurate, and understandable with regard to all aspects of care, according to the patient’s preference relative to diagnosis, prognosis, treatment, follow-up, and support services.
- Health care attends to patients’ physical symptoms and needs and provides adequate pain relief.
- Health care that addresses patients’ emotional and spiritual needs, including anxiety due to fear (e.g., the situation in which they may find themselves as victims of LEV), financial impact, or effects on family.
- Health care that involves family and friends in patient care and decision-making according to patient preference and is responsive to the needs of family and friends.

Bertini states that for any “system to be healthy, it must co-evolve with its environment; it changes in response to changes in its environment, and its environment changes in response to its changes.”^56^ While the author was speaking specifically to the transformation of the education system, the discussion above reveals that society is increasingly demanding change throughout our institutions, many of which overlap and include health care. Forensic nurses are aware and supportive of the benefits of utilizing a trauma-informed approach, as they recognize the compacting consequences of violence at every societal level.

Previously referenced were the benefits of using a patient-centered model of care that forensic nurses have understood to best manage approaches to patients who present with significant events and/or a history of trauma. However, the current “typical” hospital systems can certainly be seen as oppressive, particularly in the United States. Hegemony, “the dominance of certain ideologies, beliefs, values, or views of the world over other possible viewpoints,” guides policies and procedures that nursing and ancillary staff must adhere to while providing patient care. Because of this viewpoint, initiating change that would be beneficial to the patient and/or staff is difficult.^17^ Some hospitals now encourage nurses and staff to gather codifications “(pictures, stories and images) that represent what the people are experiencing” as an attempt to discuss processes, policies, or procedures that should change.^17^ But unless there is a demand by patients and push by staff, change will be difficult.

Hospitals are businesses, but nursing is a work of the heart that requires consideration and a personal approach. Lyckhage and colleagues highlight the context of situations such as these very well in that “all institutions are carriers and constructers of power, leading to a power order that both professionals and patients are affected by and must relate to. For nurses performing emancipatory nursing, it is important to be aware of both structural and interpersonal barriers to meet the requirements of equal care.”^22^ Across the literature, Nightingale has stated the importance of creating an ideal environment for healing and in nursing praxis with emancipatory knowledge; forensic nurses recognize that we could do better.

Patient-centered care (PCC) is necessary to facilitate positive communication between health care providers and patients. Research has demonstrated that PCC is unique to the lived experience of each patient and involves minimizing the risk of retraumatization by providing a safe psychological and physical environment.^57^ Within the umbrella of PCC, subtopics of discussion may include building trust, empowering the patient through choice and collaboration in their plan of care, practicing empathy, demonstrating cultural competence, minimizing power imbalance and consent (especially in terms of physical touch), and demonstrating a genuine desire to help the patient. The concept of self-awareness during patient interaction is unique to health care professionals. Careful consideration must be taken to avoid countertransference, maintain professional boundaries and patient-centeredness, and practice regular self-reflection.

## Patient-Centered Model of Care for Victims of LEV

**FIG. 3.**
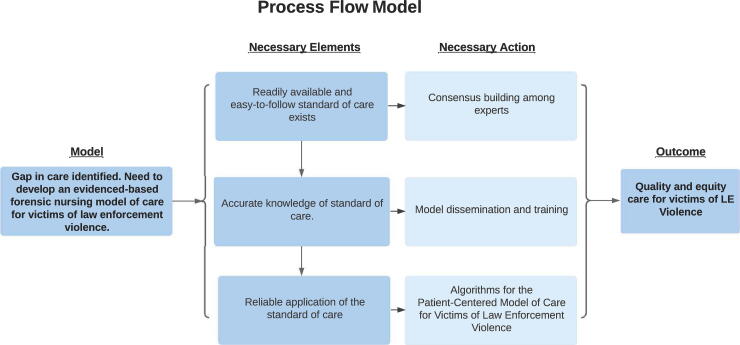
Process flow model.

Figure 3, a process flow model for developing the model of care for victims of LEV, was developed to demonstrate the primary goal driving the development of the model, necessary elements, necessary actions, and outcome expected as a result of the work, which are as follows.

Constructs from each of the models reviewed and discussed from the tenets of the Patient-Centered Model of Care for Victims of LEV. We envision “The Model” as having two distinct phases, acute and postacute, which is analogous to the care provided for victims of other types of trauma. The explanations and components of each phase are as follows.

### Acute Phase

The acute phase of care involves an initial response when patients are first evaluated by forensic nurses. Initial care may take place in a variety of settings, including emergency departments (ED), correctional, detention centers, or behavioral health settings. During this phase, the patients receive appropriate medical and forensic care in line with their injuries. As with other patients who are victims of trauma, victim advocates are also involved. As these patients have engaged in law enforcement in some way, they may be present in custody. A crucial component of the acute phase for these patients would be advocates with some knowledge of the legal system. The role of the advocate would involve guidance to keep the patient from incriminating themselves, as well as helping them understand their rights as victims. It is also essential to establish a chain of custody for handling evidence collected during forensic examinations. An additional nuance for ensuring that evidence is not mishandled is developing a process whereby the evidence is given to a law enforcement official not involved or allegedly involved as perpetrators of violence toward the patient. Patients presenting to EDs for care are generally assessed using a trauma protocol for injuries (minor or severe). Patients presenting for care in community settings would have care rendered during the normal intake process.

### Postacute Phase

**FIG. 4.**
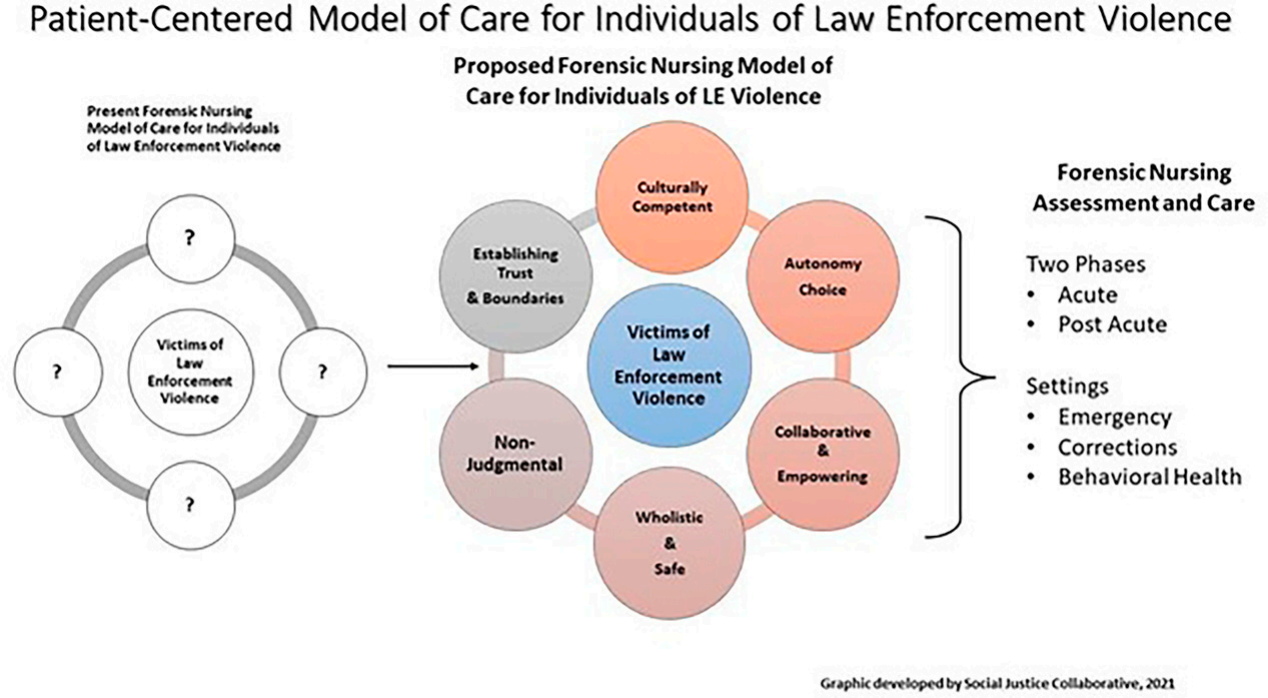
Developed by the Nursing Social Justice Collaborative, 2021.

This phase involved follow-up care and the services provided to the patient. Components included follow-up forensic examinations deemed necessary during the acute phase, ongoing advocacy, behavioral health assistance, and legal assistance analogous to the services provided to other victims of trauma. Figure 4 illustrates the lack of a present model of care for victims of LEV, constructs of the proposed model of care, and its practical applications in forensic nursing.

## Implications for Forensic Nursing Practice

Globally, forensic nursing has been understood to provide a coexistence between health care and legal systems, creating a bridge that enables resources between interdisciplinary fields to coordinate appropriate responses to those impacted by acts of violence and trauma. This relationship has proven to be challenging in terms of significant differences in approach and end goals, resulting in, as Rees describes, “a tension between medical and legal roles: a care-custody paradox.”^58^ Forensic nursing has highlighted the value of interpersonal skills, the ability to think critically, and the importance of personal maturity; however, the world over has determined the staggering differences in attitudes and behaviors between health care and law enforcement toward those caught in the crosshairs of bias, racism, low education, and discrimination.

A key element of forensic nursing education is nonjudgmental, unbiased care, which carries through the nursing profession, whether law enforcement is involved. Interprofessional collaboration dictates the awareness, role modeling, and education of forensic nursing concepts in all interactions that support patient choice and dignity.

Forensic nurses routinely encounter individuals in any setting who may have experienced LEV inclusive of but not limited to the emergency room and corrections. This standard model of care adheres to the intuitive guidelines of the International Council of Nurses, which emphasizes a nurse’s duty to care without judgment or bias, regardless of the patient’s circumstances. The following forensic nursing principles allow nurses to embrace a nontraditional role in the provision of holistic treatment, combining effective quality and practice while promoting assessment and developing improved programs of care for vulnerable individuals. Specific tasks and skill sets include assessment and treatment, as well as thorough documentation and evidence collection, as indicated by the patient’s experience. Care must be taken to follow precise forensic protocols that are strengthened in the case of LEV because of potential conflicts of interest in areas such as a chain of evidence and a departure from usual policies that may result in potential conflict with any suspected LEV perpetrator.

There may be cases in which nurses must consider whether law enforcement should occur in the examination room during the examination. According to Tahouni and colleagues, law enforcement presence during the initial assessment and care provided could pose a conflict, jeopardizing the care and safety of the patient.^59^ An individual who has been involved in LEV may perceive law enforcement presence as an ongoing threat and signal a mistrust, which extends to the health care provider. Nurses who limit law enforcement during examinations can maintain patient confidentiality while preserving the nurse–patient relationship, which is crucial in patient-centered care. For example, Canada, the United Kingdom, and Australia have policies to exclude police presence during an SA examination, which would increase undue pressure and fear of an already traumatized patient.^60–62^

The perception that Forensic Nursing is embedded within the criminal system is an inaccurate assumption. Forensic Nursing has a unique role and is positioned within the health care system to address the needs of victims to manage and restore health after trauma or injury with a trauma- and violence-informed approach.^63^ Victims rarely want forensic evidence collection; they want compassionate medical care in response to the violence they experienced. Patients want someone to listen to them. The examination of forensic evidence collection results in care following what the criminal legal system needs, not what the survivor needs. The Nursing Social Justice Collaborative believes in advocacy-driven medical forensic care. Advocacy-driven medical forensic care is related to community-based advocacy to empower victims and patients.

As in every presentation by a victim of violence, the forensic nurse has a duty beyond the provision of treatment and perhaps unequivocally more so to those who have experienced LEV due to a higher risk of connection to those who may do harm, thus risking retraumatization. Follow-up care involving several potential disciplines may be necessary to ensure that appropriate factors are considered and achieved in the provision of full patient-centered care.

## Practical Application Algorithm

**FIG. 5.**
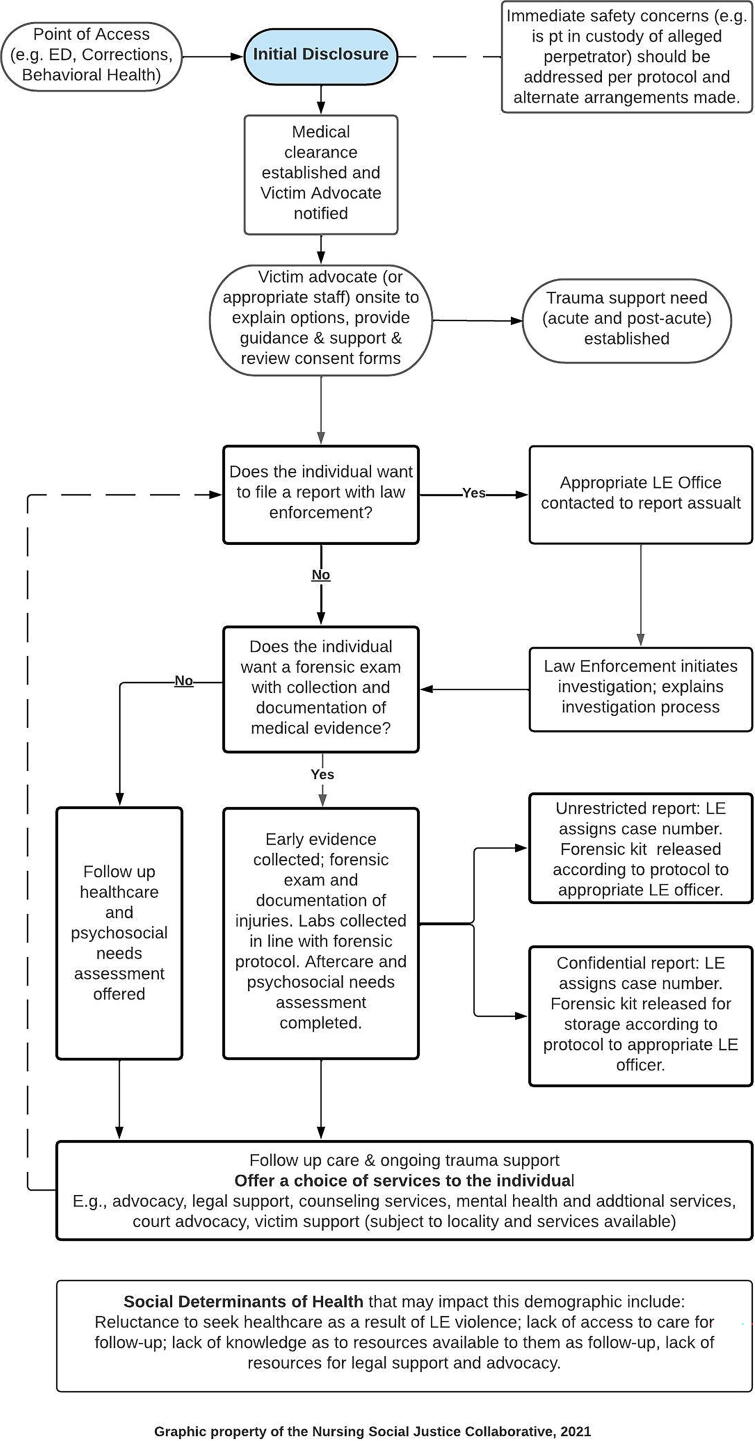
Work process algorithm: LEV victim patient care guidelines (acute and postacute). LEV, law enforcement violence.

The development of any model of care requires a discussion of practical applications. Thus, an algorithm was developed based on models of care designed for victims of other types of traumas. This algorithm provides a general demonstration of how care should be provided in acute and postacute settings. The intent of the algorithm is to provide forensic nurses working with LEV victims a guide that can be modified to fit their practice settings in global localities (Figure 5).

## Future of Forensic Nursing Care

We, the authors, and members of the Nursing Social Justice Collaborative are engaging in imagining a future for forensic nursing education, care, and practice centering on nursing care for all victims and patients of violence. Education and care must be grounded in antiracist and trauma-informed actions. Advocacy-driven medical forensic care is the future of forensic nursing care and is concerned with the narratives of those communities most disproportionately impacted by violence.^64^ Framing the structural contexts faced by patients and victims, including challenges in accessing forensic nursing care, will support forensic nursing in the necessary act of dismantling racist forensic nursing education and care. Engagement will bring light to the ideology of forensic nursing care and the stories we have told ourselves about our nursing care, center us in discomfort, examine our intentions and actions, enter a “pedagogy of discomfort,” and examine how our actions, motives, and perspectives are shaped by social structures.^65^

We understand that, currently, there is no specific model of care available to guide the care provided to individuals of LEV. In this study, we endeavored to develop a framework for this model based on the specialized care that is currently conducted by forensic nurses. We acknowledge that this newly developed model of care will continue to grow as we look to engage with other key stakeholders who also play a key role in successfully changing how these individuals are cared for after experiencing a traumatic encounter with LEV. A multidisciplinary approach is necessary and should include collaboration between health care clinicians, leadership and administration, risk management, hospital security, and other disciplines such as law enforcement and victim advocacy, all of which are essential in ensuring that there is a forensic model to be utilized when responding to coincide with the forensic nursing model of care.

## Abbreviations Used

ACEP: American College of Emergency Physicians
AMA: American Medical Association
ANA: American Nurses Association
APHA: American Public Health Association
CRT: Critical Race Theory
IAFN: International Association of Forensic Nursing
ICN: International Code of Ethics for Nurses
LEV: Law Enforcement Violence
NLN: National League for Nurses
PCC: Patient-centered care
TIC: Trauma-informed care
